# Strategies to Prevent Cardiovascular Toxicity in Breast Cancer: Is It Ready for Primetime?

**DOI:** 10.3390/jcm9040896

**Published:** 2020-03-25

**Authors:** Robin Kikuchi, Nishant P. Shah, Susan F. Dent

**Affiliations:** 1 Division of Medical Oncology, Duke Cancer Institute, Duke University, Durham, NC 27710, USA; robin.kikuchi@duke.edu; 2 Division of Cardiology, Duke Heart Center, Duke University, Durham, NC 27710, USA; nishant.shah@duke.edu

**Keywords:** Cardio-oncology, cardiotoxicity, oncology, cardiology, primary prevention, breast cancer, anthracyclines, trastuzumab

## Abstract

Cardio-oncology is an emerging field tasked with identifying and treating cancer therapy related cardiac dysfunction (e.g., cytotoxic agents, immunotherapies, radiation, and hormone therapies) and optimizing the cardiovascular health of cancer patients exposed to these agents. Novel cancer therapies have led to significant improvements in clinical outcomes for breast cancer patients. In this article, we review the current literature on assessing cardiovascular risk of breast cancer therapies and discuss strategies (including pharmacological and lifestyle interventions) to prevent cardiovascular toxicity.

## 1. Introduction

Breast cancer is the most prevalent cancer diagnosis among women in the United States, with 1 in 8 women affected [1]. Advances in cancer treatments have led to increased survivorship [1,2]. There are over 16 million cancer survivors in the United States today, and the average 5-year survival rate for women with invasive breast cancer has increased to 90% [3]. As long-term survival in adult cancer patients continues to improve, there is a need to better understand the long-term effects of cancer treatments. Clinical gains in breast cancer outcomes must be viewed in the context of potential toxicities, including cardiotoxicity. Postmenopausal breast cancer patients are at greater risk of cardiovascular (CV) mortality than breast cancer several years after their cancer diagnosis [4]. Furthermore, among breast cancer survivors, there is a greater risk of hospitalization due to cardiovascular disease (CVD) compared to the general population [5]. This is especially concerning for elderly patients who already have risk factors for CVD [5,6]. Thus, as the body of evidence regarding the importance of CV health in cancer patients has grown, the field of cardio-oncology has emerged [6,7,8].

Several breast cancer treatment modalities are known to negatively impact the CV health of patients; however, these therapies also result in a higher survivorship among breast cancer patients. The use of anthracyclines has led to significant improvements in disease-free and overall survival, as have targeted agents such as trastuzumab, in patients with human epidermal growth factor receptor positive (HER2+) breast cancer [9,10]. However, these treatments are also associated with an increased risk of heart failure, CV hospitalization, and morbidity [11,12,13]. Radiation therapy is associated with an increased risk of ischemic heart disease, particularly for those individuals who are exposed to left-sided chest wall or breast radiation where the heart is in the radiation field [14]. These CV consequences (e.g., myocardial disease, valvular disease, coronary artery disease, and vasculopathy) often appear years after radiation therapy and can persist for decades [14,15]. Additionally, the effects of endocrine therapy and early menopause can place women at an increased risk for the development of CVD as estrogen has been implicated as a cardioprotective factor in coronary heart disease [16,17]. Hormone replacement therapy has yielded mixed results with some studies suggesting increased CVD risk [18]. Additionally, downstream effects of treatment, including physical deconditioning, central obesity, increase in blood glucose, or weight gain, can increase the risk of CV dysfunction among breast cancer patients [19]. Thus, collaboration between cardiologists, oncologists, and other allied health professionals is critical to optimizing both short- and long-term CV outcomes for cancer patients.

In this article, we focus on the CV toxicity associated with anthracycline-containing regimens including those incorporating HER2-targeted therapy, which are of particular interest given their association with increased risk of cardiotoxicity. Assessment of CV risk and the role of cardioprotective strategies prior to the initiation of cancer therapy are reviewed.

## 2. Who Is at Risk?

Several position statements and guidelines have been published by the American Society of Clinical Oncologists (ASCO), the European Society for Medical Oncology (ESMO), the European Society of Cardiology (ESC), and the American Heart Association (AHA) that provide direction for identifying patients at risk of CV morbidity; however, beyond anthracyclines and trastuzumab, guidelines on other potentially cardiotoxic breast cancer therapies (e.g., cyclin-dependent kinase 4/6 inhibitors) are often based on expert opinion due to the lack of high-quality evidence.

Our current understanding suggest that CV risk factors (i.e., age, obesity, diabetes, hypertension, hyperlipidemia, comorbid renal or CVD, etc.) compounded with exposure to cardiotoxic cancer treatment increases the likelihood of CV complications [20,21,22,23,24,25,26]. Thus, screening for modifiable CV risk factors should be incorporated into a baseline risk assessment. One of those risk factors is hypertension (HTN), which can also result from anticancer therapies (radiation, cisplatin, sunitinib, etc.) [27]. In a large study from the Kaiser Permanente Southern California-SEER (Surveillance, Epidemiology, and End Results) cancer registry, HTN was more prevalent and was an independent risk factor for CV events amongst patients with cancer [28]. Dietary changes not only improve overall health but also play a big role in treating HTN in addition to medical therapy. For example, the Dietary Approaches to Stop Hypertension (DASH) diet, which is enriched with fruits, vegetables, legumes, whole grains, and low saturated fats, has been shown to effectively reduce blood pressure [29]. Good dietary habits can also help reduce cholesterol. As hyperlipidemia has been implicated in promoting cardiac inflammation, cancer patients, like all patients, should receive standard risk assessment based on the 2019 American College of Cardiology/American Heart Association (ACC/AHA) primary prevention guidelines to see if they would benefit from lipid lowering therapy [27,30]. Diabetes is another major risk factor which has been shown to increase the risk of cancer treatment-related cardiotoxicity in several studies, and it is critical to optimize management at the initiation of cancer therapy [27,31,32]. A few studies have suggested that genetic components may impact a patient’s susceptibility to developing cancer therapy-related cardiac dysfunction. (CTRCD), especially in those treated with anthracyclines. Polymorphisms in CUGBP Elav-like family member 4 and carbonyl reductases 1 and 3 have been implicated in increased anthracycline-related cardiotoxicity even at low doses [33,34]. Although genetic screening is not suggested in any guideline, the role of genetics may explain why some patients develop cardiotoxicity and some do not and why no dose of anthracyclines should be considered safe. Further research into the role of genetics in CTRCD is needed.

The role of cardiac imaging in the early detection of CTRCD continues to evolve. Multimodality cardiac imaging such has echocardiography, cardiac computed tomography (CT), and/or cardiac magnetic resonance imaging (MRI) can be helpful in defining those patients at greatest risk of CTRCD at baseline (e.g., low left ventricular ejection fraction (LVEF) at baseline) and can contribute to decision making with regards to optimal cancer therapy [35,36,37,38,39]. Multigated acquisition imaging, although still used in oncology practice, is no longer recommended due to the associated exposure to radiation [27]. Global longitudinal strain imaging (GLS) is the recommended measurement of myocardial deformation due to its high sensitivity for early detection of left ventricular (LV) dysfunction. Thus, GLS imaging at baseline using doppler tissue imaging or 2D speckle tracking echocardiography is recommended [40]. Imaging prior to treatment allows for the identification of any preexisting cardiac concerns as well as establishes a baseline should any functional changes occur during treatment [2,27]. ESMO recommends that patients receiving anthracyclines have baseline imaging and then after a cumulative dose of 250mg/m^2^ and every 100mg/m^2^ thereafter. Patients receiving HER2-targeted therapy should have cardiovascular imaging every 3 months during treatment; survivors should be screened at 6–12 months, 2 years, and periodically thereafter [27]. Due to variability in cardiac imaging recommendations and limited supporting evidence, some patients may undergo unnecessary cardiac imaging (e.g., healthy women with breast cancer receiving adjuvant trastuzumab) while others may not have adequate cardiac imaging (e.g., strong history of heart disease and metastatic breast cancer on HER2-targeted therapy indefinitely) to prevent cardiovascular decompensation. Further research is needed to develop personalized imaging strategies for cancer patients exposed to potentially cardiotoxic therapy.

In those who have developed structural cardiac disease, biomarkers such as cardiac troponin and brain natriuretic peptide (BNP) have been shown to help prognosticate and have been suggested for use in patients most at risk of developing CV complications [27,41,42]. Cardinale et al., in a study of 251 women with breast cancer receiving trastuzumab, demonstrated that troponin I levels were the only independent predictor of both treatment induced cardiotoxicity (HR, 22.9; 95% CI, 11.6 to 45.5; *p* < 0.001) and lack of LVEF recovery (HR, 2.88; 95% CI, 1.78 to 4.65; *p* < 0.001) [41]. Another novel approach to CV risk stratification may be through the measurement of breast arterial calcification (BAC) seen on routine mammography. In a study of 278 women, BAC was independently associated with the presence or development of heart failure, suggesting BAC as a marker of increased CVD risk. Further study of this novel method is underway [43,44].

There have been attempts to predict the risk CTRCD. Romond et al. developed a prediction tool using follow-up data from the National Surgical Adjuvant Breast and Bowel Project B-31. They identified age and baseline LVEF as predictors of CV toxicity [22]. Ezaz et al. derived a comprehensive ranking system tool using data from the US Surveillance, Epidemiology, and End Results database. They identified age, adjuvant chemotherapy, history of cardiac disease, and cardiac risk factors as predictors of CV toxicity based on the database [23]. This tool demonstrated good specificity and negative predictive value in a real world cohort of 143 breast cancer patients [45]. Using administrative databases in Ontario, Canada, Abdel-Qadir et al. developed the most comprehensive tool to date, identifying age, HTN, diabetes, ischemic heart disease, atrial fibrillation, cerebrovascular disease, heart failure, peripheral vascular disease, chronic obstructive pulmonary disease, and chronic kidney disease as predictors of major CV events during treatment. The agreement between predicted and observed CV incidence was strong in the validation cohort. They validated the tool with a c-index of 81.9% (95% CI, 80.9% to 82.9%) at 5 years and 79.8% (95% CI, 78.8% to 80.8%) at 10 years [46]. These multivariable risk score tools have generally not been adopted into clinical practice: they require further validation, are still fairly simplistic, and do not incorporate variables such as biomarkers or advanced cardiac imaging [21,22,23,47]. Several organizations, such as the International Cardio Oncology Society (ICOS), have attempted to establish more widespread databases with the hope of creating a more comprehensive and predictive risk score. However, ICOS as well as other organizations have struggled to establish such databases due to limited funding. Ultimately, routine baseline screening will facilitate early intervention to mitigate cardiotoxicity in those patients most at risk. Thus, methods to decrease CV risk either through lifestyle modifications or pharmacotherapy prior to initiation of cancer therapy are important to consider.

## 3. Primary Prevention Strategies

Our improvement in understanding the CV consequences of cancer therapy has led to increasing interest in exploring prevention of CTRCD. Several randomized trials have explored primary prevention strategies in breast cancer patients receiving anthracycline containing regimens, with or without trastuzumab.

Dexrazoxane, a derivative of ethylenediaminetetraacetic acid (EDTA) that works as an iron chelator to reduce the formation of active anthracycline metabolite complexes that lead to oxidative stress, has been studied as a cardioprotective agent both in the pediatric and adult cancer population [48]. Dexrazoxane reduces the formation between topoisomerase Iiβ and anthracyclines to help prevent cardiotoxicity [48]. The agent was first tested in patients with advanced breast cancer receiving anthracyclines in the 1990′s and was found to have a significant reduction in cardiotoxicity, defined as clinical signs of congestive heart failure, a decrease in absolute LVEF to ≤45%, or a decrease in LVEF of ≥20% (OR 0.29 (95% CL 0.09–0.78; *p* = 0.06) [49]. Dexrazoxane is approved by the United States Food and Drug Administration (FDA) for use in advanced breast cancer patients treated with anthracyclines who have reached a cumulative dose of 300 mg/m^2^. Today, dexrazoxane remains the only FDA-approved cardioprotective agent for anthracycline-containing regimens. The American Society of Clinical Oncology endorsed the use of dexrazoxane in patients with advanced breast cancer scheduled to receive high-dose anthracycline treatment [2]; however, there continues to be concern for the development of secondary malignancies and reduction in antitumor activity. Thus, clinicians have been hesitant to adopt this approach in clinical practice [50]. However, a recent systematic review of seven randomized clinical trials using dexrazoxane in breast cancer patients treated with and without trastuzumab found no significant impact on cancer outcomes [48]. Despite these findings, the authors concluded that the quality of evidence was low and that further research was needed. Another strategy to reduce anthracycline toxicity is using continuous infusion rather than bolus administration of anthracycline-containing chemotherapies [51]. Administration of weekly divided doses of anthracyclines significantly decreases CV damage without compromising the tumor response rate or overall survival of patients [51,52]. However, these methods of administration often require a central line which comes with risks and frequent visits which can be cumbersome for patients. Additionally, less cardiotoxic anthracyclines such as liposomal doxorubicin have been studied in patients to prevent cardiotoxicity. In liposomal doxorubicin, the active doxorubicin is encased in a liposomal covering, allowing the drug to more readily penetrate the more porous tumor tissue without the same level of exposure to other organs as with other anthracycline-based treatment modalities [51,53]. However, due to high costs, liposomal doxorubicin has not been widely implemented and is not currently approved for the treatment of breast cancer [51,54].

### 3.1. ACE Inhibitors, Angiotensin Receptor Blockers, and Beta-Blockers

There are several studies investigating the cardioprotective efficacy of ACE inhibitors (ACEI), angiotensin receptor blockers (ARB), and beta-blockers for breast cancer patients treated with anthracycline-containing treatment modalities (Table 1). In the CECCY (Carvedilol Effect in Preventing Chemotherapy-Induced Cardiotoxicity) trial, 200 HER2 patients receiving treatment with doxorubicin were randomized in a 1:1 ratio to receive either the beta-blocker carvedilol or placebo. The primary endpoint was to measure LVEF from baseline to 6 months after the initiation of chemotherapy with ≥10% decline considered to be indicative of CV dysfunction. The results were not statistically significant (*p* = 1.00), with 14.5% in the carvedilol group and 13.5% in the placebo group experiencing LVEF decline of >10% [55]. Boekhout et al. conducted a trial of 200 HER2+ patients all receiving treatment with epirubicin-based therapy followed by trastuzumab randomized in a 1:1 ratio to receive either the ARB candesartan or placebo. The primary endpoint was to measure cardiac dysfunction among patients defined for this study as an LVEF decline of greater than 15% or a decrease below the absolute value of 45%. Their findings were also not statistically significant (*p* = 0.58), with 19% of the candesartan group and 16% of the placebo group experiencing cardiac dysfunction [56].

Three studies have published promising findings on the efficacy of these treatment modalities. The PRADA (Prevention of Cardiac Dysfunction During Adjuvant Breast Cancer Therapy) study randomized 130 early stage breast cancer patients receiving anthracycline-containing chemotherapies, 22% of which were also receiving trastuzumab, to receive the beta-blocker metoprolol, the ARB candesartan, both, or the placebo in a 1:1:1:1 ratio. The outcome was measured as changes in LVEF measured by cardiac magnetic resonance (CMR) imaging between 10 and 64 weeks. The results of candesartan were considered significant (*p* = 0.026) with 0.8% change in LVEF as compared to 2.6% in the placebo group [57]. The MANTICORE (Multidisciplinary Approach to Novel Therapies in Cardio-Oncology Research) trial randomized 94 HER2+ patients being treated with trastuzumab, 33% of which were receiving anthracycline-containing chemotherapy treatment, in a 1:1:1 ratio to receive the beta-blocker bisoprolol, the ACEI perindopril, or the placebo. The primary outcome was cardiac remodeling measured as change in indexed left ventricular end diastolic volume on cardiac MRI, and the secondary outcome was the change in LVEF on cardiac MRI at baseline and 1 year. Patients receiving bisoprolol experienced an average decline in LVEF of 1%, vs. 3% in those receiving perindopril and 5% for those taking placebo. The results for both treatment modalities were considered significant (*p* = 0.001) [58]. Lastly, the largest trial conducted by Guglin et al. studied 468 HER2+ patients receiving trastuzumab, 40% of which were concurrently being treated with doxorubicin, randomized in a 1:1:1 ratio to receive the beta-blocker carvedilol, the ACEI lisinopril, or the placebo. Patient were evaluated over two years with LVEF >10% or LVEF decline >5% with absolute LVEF < 50% considered to be indicative of cardiotoxicity. Among the population with prior anthracycline exposure, 31% receiving carvedilol, 37% receiving lisinopril, and 47% receiving the placebo experienced clinically significant LVEF decline. These results were considered statistically significant (*p* = 0.009) [59].

These were small studies conducted in relatively young, healthy patients with few comorbidities [55,56,57,58,59]. These trials had different definitions of cardiotoxicity, used different cardiac medications, and had short duration of follow-up, limiting the generalizability of the findings (Table 1). The clinical benefit of preventing small declines in LVEF in asymptomatic patients is not clear. Thus, while ACEIs, ARBs, and beta-blockers are promising, additional large-scale trials are needed, ideally in higher risk populations, before they are recommended as a standard of care for cancer patients who are exposed to potentially cardiotoxic cancer therapy. Longer duration of follow-up and clinically meaningful clinical endpoints (e.g., development of heart failure) will further strengthen this approach.

Cardioprotective agents have also been studied in patients with mild cancer therapy-related cardiac dysfunction. A recently published study by Geuna et al. explored the potential efficacy of ACEIs and beta-blockers as a secondary prevention strategy with promising results; 103 women being treated with adjuvant trastuzumab were enrolled. Those who developed mild cardiotoxicity (*n* = 16) were treated with enalapril and carvedilol. Geuna et al. demonstrated that patients post-trastuzumab treated with study drugs were able to achieve the same LVEF values of patients that did not develop cardiotoxicity. However, more large-scale inquiries must be carried out to verify these findings [60].

### 3.2. Statins

Statins are another class of drugs that have been evaluated for their cardioprotective role in cancer patients exposed to cardiotoxic agents. Findings in mice receiving doxorubicin and statins have been promising for mitigating cardiotoxicity [61]. Seicean et al. published a retrospective study of 628 women with newly diagnosed breast cancer treated with anthracyclines. Women in this study varied in age and level of CV comorbidity at baseline; 17.2% had HTN, and 5.1% had diabetes. Of the participants, 67 receiving uninterrupted statin therapy throughout the approximately 3-year follow-up period demonstrated significantly lower hazard ratios (HR: 0.3; 95% CI: 0.1 to 0.9; *p* = 0.03) for development of heart failure as compared to the control groups [62]. The decreased valvular inflammation and oxidative stress associated with the pleiotropic effects of statins are theorized to mitigate the effects of cardiotoxicity; however, the exact mechanisms of the effect of statins on cardiotoxicity remains largely unknown [62]. More studies regarding the use of statins are emerging in the field of cardio-oncology, and further trials are needed to determine efficacy and possible combination therapies.

### 3.3. Mineralocorticoid Receptor Antagonists

Mineralocorticoid receptor antagonists, such as spironolactone, in addition to ACEI, ARB, or beta-blocker therapy, has been shown to reduce CV mortality in heart failure patients with reduced ejection fractions [63]. They are thought to lead to a reduction in fibrosis, extracellular matrix turnover, and myocardial collagen content, thus affecting the progression of HF and improving LV function [64]. Therefore, mineralocorticoid receptor antagonists have become a new area of inquiry for the primary prevention of anthracycline-induced cardiotoxicity. Akpek et al. conducted a trial of 83 breast cancer patients randomized in a 1:1 ratio to 25 mg of spironolactone per day vs. placebo. They found that the interaction of LVEF decrease between groups was significantly lower in the spironolactone group than the control group (*p* < 0.001) [65]. Further, large-scale research is needed to confirm the benefit observed in this study.

### 3.4. Lifestyle Modifications

Despite advances in research for cardioprotective drugs, the impact of lifestyle modifications cannot be understated. The diagnosis of cancer in itself can lead to depression, sedentary behavior, loss of functional capacity, and weight gain. Studies have shown fewer CV events in cancer patients who exercise on a regular basis. In a prospective analysis of 55 females with breast cancer who received anthracycline therapy, were 65 or younger, and had no CV risk factors, there were fewer symptoms of heart failure in physically active patients over a 5-year follow-up period [66]. There was also a delayed onset of diastolic dysfunction compared to inactive patients [66]. In another, larger prospective analysis of 2973 nonmetastatic breast cancer participants with an 8.6 year median follow-up, routine exercise of ≥9 MET-hour/week was associated with a reduction in CV events (new diagnosis of coronary artery disease, valve abnormality, arrhythmia, stroke, or CVD death) [67]. This relationship was also graded based on the level of activity [67]. Furthermore, there is data suggesting those who start physical activity after a diagnosis of breast cancer have a lower risk of death in general (HR 0.33 (95% CI 0.15–0.73; *p* = 0.046)) [68]. Thus, the findings of these studies have prompted the American Heart Association (AHA) to recommend physical activity as an individualized cardiac rehabilitation intervention for those most at risk of developing cardiotoxicity [69] and the European Society for Medical Oncology (ESMO) to recommend exercise for all cancer survivors [27]. However, further large-scale inquiry is warranted to determine specific exercise recommendations based on population and risk stratification.

Overall, the data for the use of conventional as well as lifestyle-based CV treatments to prevent cardiotoxicity in breast cancer patients prescribed anthracyclines and/or HER2-targeted therapies is promising. Larger studies, particularly in at-risk populations, with longer follow-up are needed to determine if these therapies remain effective long term and to determine the optimal dosage and duration of cardioprotective therapy. Additionally, future studies should focus on defining the best cardioprotective strategies in patients exposed to novel cancer drugs (e.g., immunotherapy) to define best clinical practices. Studies exploring combining two or more cardioprotective agents as well as other cardiac drugs should be conducted since agents can have varying cardioprotective effects [70]. Lastly, studies need to focus on more diverse populations as the majority of studies have focused on predominantly young patients with few comorbidities. Studies of patients with CV comorbidities as well as those in patients with advanced cancer are critical to determine the best strategies for these high risk populations.

## 4. Future Directions

Although the current literature yields promising results suggesting cardioprotective strategies that can be incorporated into treatment plans on an individualized basis, it is not definitive in terms of efficacy, optimal dosage, and combination therapies for primary prevention strategies of CV toxicity. Several studies, both pharmacological and lifestyle based, are being conducted that should provide further evidence to support this approach.

There are several ongoing studies investigating pharmacology-based interventions (Table 2). PROACT (Preventing Cardiac Damage in Patients Treated for breast Cancer: A Phase 3 Randomized, Open Label, Blinded Endpoint, Superiority Trial of Enalapril to Prevent Anthracycline-induced CardioToxicity) (NCT03265574) is currently investigating enalapril as a primary prevention strategy amongst postsurgical breast cancer patients treated with epirubicin. The primary outcome is measuring troponin T release during epirubicin treatment. Additionally, ICOS-ONE (The International CardioOncology Society-One Trial) (NCT01968200) is investigating the efficacy of enalapril as a cardioprotective agent in a variety of cancer patients receiving anthracyclines. The primary outcome measurement is the occurrence of cardiac troponin elevation above the defined threshold during treatment. The Southwest Oncology Group (SWOG) S1501 trial (NCT03418961) is a randomized phase III trial investigating the efficacy of the beta-blocker carvedilol versus no intervention in metastatic HER2+ breast cancer patients receiving potentially cardiotoxic treatments. The primary outcome is the time to the first indication of cardiac dysfunction as measured by changes in LVEF as indicated by echocardiogram. Another study, the Cardiac CARE (The Cardiac CARE Trial—can heart muscle injury related to chemotherapy be prevented?) trial (ISRCTN24439460), is investigating the efficacy of beta-blockers in addition to ARBs in breast cancer patients with increased troponin levels receiving anthracyclines as measured by magnetic resonance imaging (MRI) at 6 months. The PRADA (Prevention of Cardiac Dysfunction During Adjuvant Breast Cancer Therapy) II study (NCT03760588), led by the same team that carried out the original PRADA study, is investigating entresto, a combination medication for heart failure that combines a neprilysin inhibitor and an ARB. Changes in LVEF is the primary outcome as measured by MRI at 18 months.

Research investigating the clinical benefit of statins is ongoing. STOP-CA (Statins to Prevent the Cardiotoxicity from Anthracyclines) (NCT02943590) is evaluating the efficacy of statins as a primary prevention strategy amongst non-Hodgkin’s Lymphoma patients receiving anthracycline containing regimens. The primary outcome is changes in LVEF as measured by MRI and echocardiography at 12 months. PREVENT (Preventing Anthracycline Cardiovascular Toxicity with Statins) (NCT01988571) is investigating the efficacy of the statin atorvastatin amongst both breast cancer and lymphoma patients receiving anthracyclines. The primary outcome is preservation of LV function as measured by MRI, biomarkers, and symptoms at 2 years.

There are also several ongoing studies (NCT02244411, NCT03223753, NCT03104543, and NCT03386383) exploring web-based diet and exercise interventions in cancer survivors that could have implications for lifestyle-based prevention strategies both during treatment and into survivorship (Table 2). Specifically, two trials (NCT02244411 and NCT03104543), exploring the efficacy of care plans that encompass both diet and exercise interventions, are evaluating patients with comorbid CV risk factors such as obesity. This could result in reduced CTRCD, increased understanding of the effects of CV comorbidity, and better patient outcomes in quality of life and overall health. Additionally, the CARDAPAC (Physical Activity Intervention on Myocardial Function in Patients with HER2 + Breast Cancer) (NCT02433067) study is exploring the impact of individualized exercise interventions (45 min, 3 days a week) in breast cancer patients treated with trastuzumab on LVEF, body composition, muscle function, metabolic, hormonal and inflammatory responses, pain, fatigue, and quality of life. The results of these studies will help affirm the efficacy and practical applications of these treatment strategies in breast cancer patients receiving anthracycline and other cardiotoxic therapies.

## 5. Conclusions

In the field of cardio-oncology, an important emerging area of research is the prevention of CTRCD. CV risk factors should be evaluated at the time of cancer diagnosis and considered when deciding upon cancer treatment and CV monitoring strategies for the best patient outcomes. In patients at higher CV risk, a multipronged approach and comprehensive health care plan should be considered to reduce the risk of CTRCD. Cancer therapy can be modified or combined with pharmacological and/or lifestyle interventions to improve patient outcomes. The current pharmacological interventions (e.g., ACEI, ARB, and BB) may not be ready for widespread application in all patients, as further studies are needed to determine the efficacy and optimal duration and dosage of these interventions as well as their impact in more diverse patient populations. These cardioprotective strategies, however, are being incorporated into practice by clinicians for those patients identified as most at risk, in keeping with the most recent ESMO consensus [27].

The role of cardiac imaging and the incorporation of biomarkers to identify and manage those patients at greatest risk of CTRCD continues to evolve [2,27]. Early detection of changes in CV health prior to treatment will improve patient outcomes through tailored comprehensive health plans and the timely application of cardioprotective strategies.

As data continues to emerge, increasingly clear strategies for the prevention and management of CV toxicity among cancer patients should continue to be a major area of inquiry. Ultimately, further research on preventative strategies across solid and hematological malignancies is needed as the majority of primary prevention trials have been in breast cancer populations. The goal of clinicians should be to develop strategies to provide optimal cancer therapy while minimizing any potential detrimental impact on CV health.

## Figures and Tables

**Table 1:** Primary cardiotoxicity prevention trials in patients with breast cancer.

Trial (N)	Intervention	Primary Outcome	Benefit (Yes/No)
PRADA [57] (130, all anthracycline, 22% trastuzumab)	1:1:1:1, metoprolol, candesartan, metoprolol and candesartan, or placebo	Changes in LVEF by CMR at 10 to 64 weeks	Yes, absolute LVEF change: 2.6% in placebo, 0.8% in candesartan (*p* = 0.026)
MANTICORE [58] (94, all trastuzumab, 12–33% anthracycline)	1:1:1 bisoprolol, perindopril, or placebo	Changes in LV end diastolic volume Indexby CMR at 1 year	Yes, small reduction in LVEF decline with bisoprolol compared with perindopril and placebo (–1% vs. –3% vs. –5%, *p* = 0.001)
Guglin et al. [59] (468, all trastuzumab, 40% doxorubicin)	1:1:1 carvedilol, lisinopril, or placebo	LVEF > 10% or LVEF decline > 5% with absolute LVEF < 50%	Yes, >10% LVEF decline in subset with prior anthracycline exposure: 47% placebo, 31% carvedilol, 37% lisinopril (*p* = 0.009)
CECCY [55] (200, all doxorubicin)	1:1 carvedilol or placebo	LVEF > 10% decline from baseline to 6 months	No, LVEF decline: 13.5% placebo,14.5% carvedilol (*p* = 1.00)
Boekhout et al. [56] (206, all epirubicin with trastuzumab)	1:1 candesartan or placebo	LVEF decline of >15% or a decrease below the absolute value of 45%	No, LVEF decline: 19% in candesartan, 16% in placebo (*p* = 0.58)

LVEF: Left Ventricular Ejection Fraction; LVEDI: Left Ventricular End Diastolic Volume Index; CMR: Cardiac Magnetic Resonance; PARDA: Prevention of Cardiac Dysfunction During Adjuvant Breast Cancer Therapy; MANTICORE: Multidisciplinary Approach to Novel Therapies in Cardio-Oncology Research; CECCY: Carvedilol Effect in Preventing Chemotherapy-Induced Cardiotoxicity.

**Table 2:** Ongoing pharmacological and lifestyle cardioprotection trials.

Trial Name (PI)	Trial Intervention	Population	Primary Outcomes
**Pharmacological Interventions**			
PREVENT (NCT01988571) (Hundley, Wake Forest)	Statins (Atorvastatin) vs. Placebo	Breast cancer, lymphoma, chemotherapy with anthracyclines	MRI, biomarkers, symptoms at 2 years
STOP-CA (NCT02943590) (Neilan, Scherrer Crosbie, MGH)	Statins vs. Placebo	Non-Hodgkin’s Lymphoma, chemotherapy with anthracyclines	MRI, echo at 12 months
SWOG S1501 (NCT03418961) (Floyd, Leja, Fabian, SWOG)	Carvedilol vs. No Intervention	Metastatic HER2+ breast cancer	Time to first indication of cardiac dysfunction as measured by changes in LVEF by echo
ICOS-ONE (NCT01968200) (Cipolla, European Institute of Oncology)	Enalapril preventatively vs. After indication of cardiotoxicity	Cancer, chemotherapy with anthracyclines	Cardiac troponin level elevation above threshold
PROACT (NCT03265574) (Change, Newcastle University)	Enalapril vs. No Intervention	Post-surgical breast cancer to be treated with epirubicin	Cardiac troponin T release during epirubicin treatment
CARDIAC CARE (ISRCTN24439460) (Maclean, The Queen’s Medical Research Institute)	Angiotensin Receptor Blockers and B-blocker or No Intervention	Breast cancer, chemotherapy with anthracyclines, increased cardiac troponin levels	MRI at 6 months
PRADA II (NCT03760588) (Omland, Gulati)	Entresto vs. Placebo	Breast cancer, chemotherapy with anthracyclines	Left ventricular ejection fraction by cardiovascular magnetic resonance at 18 months
**Lifestyle Interventions**			
EQUAL (NCT02244411) (Tonorezos)	Web-based diet and activity intervention	Adult aged, obese survivors of childhood acute lymphoblastic leukemia	Weight loss at 2 years
NCT03223753 (Ness)	Web-based physical activity intervention	Childhood acute lymphoblastic leukemia patients within 3 months of completing therapy	Physiologic cost index at 6 months
NCT03104543 (Chow)	Survivorship care plan counseling intervention	Adult-aged survivors at high risk of cardiovascular disease	Blood pressure, cholesterol, glucose, and lipids at 1 year
NCT03386383 (Valle)	Mobile intervention with tailored feedback	Young adult (18–39) cancer survivors	Physical activity (ActiGraph)
CARDAPAC (NCT02433067) (Mougin-Guillaume)	Three months individualized physical activity (45 min, 3 times per week)	HER2+ breast cancer patients treated only by trastuzumab	LVEF, body composition, muscle function, metabolic, hormonal and inflammatory responses, pain, fatigue, quality of life

PREVENT: Preventing Anthracycline Cardiovascular Toxicity with Statins; STOP-CA: Statins to Prevent the Cardiotoxicity from Anthracyclines; SWOG: Southwest Oncology Group; ICOS-ONE: The International CardioOncology Society-One Trial; PROACT: Preventing Cardiac Damage in Patients Treated for breast Cancer: A Phase 3 Randomized, Open Label, Blinded Endpoint, Superiority Trial of Enalapril to Prevent Anthracycline-induced CardioToxicity; CARDIAC CARE: The Cardiac CARE Trial—can heart muscle injury related to chemotherapy be prevented?; EQUAL: Exercise and Quality Diet After Leukemia: The Equal Study; CARDAPAC: Physical Activity Intervention on Myocardial Function in Patients with HER2 + Breast Cancer.
